# Thrombolysis in Stroke With Unknown Onset Based on Non‐Contrast Computerized Tomography (TRUST CT)

**DOI:** 10.1161/JAHA.119.014265

**Published:** 2020-02-12

**Authors:** Marek Sykora, Lars Kellert, Patrik Michel, Ashraf Eskandari, Katharina Feil, Jan Rémi, Julia Ferrari, Stefan Krebs, Wilfried Lang, Wolfgang Serles, Pavel Siarnik, Peter Turcani, Michal Kovacik, Benjamin Bender, Annerose Mengel, Khouloud Poli, Sven Poli

**Affiliations:** ^1^ Department of Neurology St. John's Hospital Medical faculty Sigmund Freud University Vienna Austria; ^2^ Department of Neurology Ludwig Maximilians University Munich Germany; ^3^ Stroke Center, Neurology Service Department of Clinical Neurosciences Lausanne University Hospital Lausanne Switzerland; ^4^ German Center for Vertigo and Balance Disorders Ludwig Maximilians University Munich Germany; ^5^ Department of Neurology Medical University Vienna Austria; ^6^ Department of Neurology Comenius University Bratislava Slovakia; ^7^ Department of Neurology General Hospital Liptovsky Mikulas Slovakia; ^8^ Department of Diagnostic and Interventional Neuroradiology University Hospital Tübingen Germany; ^9^ Department of Neurology with Focus on Neurovascular Diseases and Neurooncology and Hertie Institute for Clinical Brain Research University Hospital Tübingen Germany

**Keywords:** alteplase, CT, outcome, safety, thrombolysis, wake‐up stroke, Cerebrovascular Disease/Stroke, Ischemic Stroke, Cerebrovascular Procedures

## Abstract

**Background:**

Intravenous thrombolysis (IVT) in wake‐up stroke (WUS) or stroke with unknown onset (SUO) has been recently proven to be safe and effective using advanced neuroimaging (magnetic resonance imaging or computerized tomography‐perfusion) for patient selection. However, in most of the thrombolyzing centers advanced neuroimaging is not instantly available. We hypothesize that pragmatic non‐contrast computed tomography‐based IVT in WUS/SUO may be feasible and safe.

**Methods and Results:**

TRUST‐CT (Thrombolysis in Stroke With Unknown Onset Based on Non‐Contrast Computerized Tomography) is an international multicenter registry‐based study. WUS/SUO patients undergoing non‐contrast computed tomography‐based IVT with National Institute of Health Stroke Scale ≥4 and initial Alberta Stroke Program Early Computerized Tomography score ≥7 were included and compared with propensity score matched non‐thrombolyzed WUS/SUO controls. Primary end point was the incidence of symptomatic intracranial hemorrhage; secondary end points included 24‐hour National Institute of Health Stroke Scale improvement of ≥4 and modified Rankin Scale at 90 days. One hundred and seventeen WUS/SUO patients treated with non‐contrast computed tomography‐based IVT were included. As compared with 112 controls, the median admission National Institute of Health Stroke Scale was 10 and the median Alberta Stroke Program Early Computerized Tomography score was 10 in both groups. Four (3.4%) IVT patients and one control patient (0.9%) suffered symptomatic intracranial hemorrhage (adjusted odds ratio 7.9, 95% CI 0.65–96, *P*=0.1). A decrease of ≥4 National Institute of Health Stroke Scale points was observed in 67 (57.3%) of IVT patients as compared with 25 (22.3%) in controls (adjusted odds ratio 5.8, CI 3.0–11.2, *P*<0.001). A months, 39 (33.3%) IVT patients reached a modified Rankin Scale score of 0 or 1 versus 23 (20.5%) controls (adjusted odds ratio 1.94, CI 1.0–3.76, *P*=0.05).

**Conclusions:**

Non‐contrast computed tomography‐based thrombolysis in WUS/SUO seems feasible and safe and may be effective. Randomized prospective comparisons are warranted.

**Clinical Trial Registration:**

URL: https://www.clinicaltrials.gov/. Unique identifier: NCT03634748.

**Clinical Perspective**

**What Is New?**

- Non‐contrast computed tomography‐based intravenous thrombolysis in wake‐up stroke or stroke with unknown onset seems feasible, safe, and may be effective.

**What Are the Clinical Implications?**

- If confirmed in a randomized prospective manner, the non‐contrast computed tomography ‐based approach will make thrombolytic therapy accessible to patients with wake‐up stroke or stroke with unknown onset also in the absence of computerized tomography‐perfusion or magnetic resonance imaging.

## Introduction

Intravenous thrombolytic therapy (IVT) for ischemic stroke was repeatedly proven safe and effective in the time window of 4.5 hours after symptoms onset.^1^, ^2^ Wake‐up stroke (WUS) and stroke with unknown onset (SUO) have been traditionally excluded from IVT because of missing information on the time since symptoms occurred. Despite sharing similar clinical and radiological features with known onset stroke, IVT in WUS/SUO was not proven effective and safe until recently.^3^ Published in 2018, the randomized controlled WAKE‐UP trial successfully introduced a radiological paradigm using magnetic resonance imaging and the fluid‐attenuated inversion recovery/diffusion weighted imaging mismatch for selection of WUS patients eligible for IVT.^4^ Even more recently, the EXTEND randomized controlled trial positively proved the role of computerized tomography (CT)‐perfusion mismatch in wake‐up stroke IVT.^5^ However, the majority of centers administering IVT do not have 24/7 access to advanced neuroimaging (magnetic resonance imaging or CT‐perfusion). Here, we aimed to test a pragmatic and potentially widely applicable approach that does not require advanced neuroimaging to determine eligibility for IVT in WUS/SUO. Previous retrospective case series and monocentric, single‐arm studies suggested feasibility and safety of using non‐contrast computed tomography (NCCT) for selecting WUS/OUS patients for IVT.^6^, ^7^, ^8^, ^9^ However, because of the limited numbers of subjects included and a non‐controlled design, the evidence is scarce. We hypothesize that NCCT‐based IVT using Alberta Stroke Program Early CT score^10^ in WUS/SUO patients is feasible, safe, and eventually effective.

## Methods

The data that support the findings of this study are available from the corresponding author upon reasonable request.

### Design

Pre‐defined retrospective analysis of a dedicated, prospective, multicenter, international registry including WUS/OUS patients undergoing standard IVT with 0.9 mg/kg recombinant tissue plasminogen activator rtPA solely based on NCCT appearance.

### Study Population

Consecutive WUS/OUS patients treated with IVT at 5 European stroke centers (Department of Neurology, St. John's Hospital Vienna; Austria, Department of Neurology, University Tuebingen; Germany, Department of Neurology, Ludwig Maximilians University, Munich, Germany; Department of Neurology, Comenius University Bratislava, Slovakia; and Department of Neurology, General Hospital, Liptovsky Mikulas, Slovakia) between September 1, 2017 and December 31, 2018 were recorded, if the inclusion and exclusion criteria were fulfilled, see Table 1. The time from last seen well had to be >4.5 hours and the time from symptom discovery to hospital arrival had to be <4.5 hours. Patients with CT perfusion studies or MR imaging before IVT were excluded. Patients undergoing IVT and additional endovascular thrombectomy because of large vessel occlusion were included.

**Table 1:** TRUST CT Study: Inclusion and Exclusion Criteria

Inclusion criteria Ischemic stroke with NIHSS ≥4; Wake‐up stroke or stroke with unknown onset with last seen well >4.5 h; Aged ≥18 y; IVT started within 4.5 h of awakening and/or within 4.5 h of discovering symptoms; IVT started within 30 min of admission CT; and Non‐contrast CT scan with no early signs or early signs equivalent to ASPECTS ≥7 Exclusion criteria Non‐contrast CT scan with clear hypodensity or early signs equivalent to ASPECTS ≤6; Evidence of intracranial or subarachnoid hemorrhage; Inability to control high systolic blood pressure >185 mm Hg, or high diastolic blood pressure >110 mm Hg with intravenous antihypertensive medication; Known coagulopathy or evidence of active bleeding; Surgical procedures, subclavian arterial puncture, trauma, and gastrointestinal or genitourinary bleeding within 14 d of the event; Patients taking direct oral anticoagulants within last 48 h; Patients taking vitamin K antagonists and having an INR >1.7; Platelet count <100 000 per μL, venous glucose either <50 or >450 mg/dL, or Pre‐stroke modified Rankin Scale score >3

ASPECTS indicates Alberta Stroke Program Early Computerized Tomography Score; CT, computed tomography; INR, international normalized ratio; IVT, intravenous thrombolysis; NIHSS, National Institute of Health Stroke Scale; TRUST CT, Thrombolysis in Stroke With Unknown Onset Based on Non‐Contrast Computerized Tomography.

### Clinical Data

Local sites collected the following demographic and clinical data: age, times of last seen well and symptom discovery, hospital arrival, and door‐to‐needle time, initial Alberta Stroke Program Early CT score (ASPECTS),^10^ admission NIHSS score, pre‐stroke modified Rankin Scale (mRS), medical history of previous stroke/TIA, hypertension, diabetes mellitus, atrial fibrillation, coronary artery disease, hyperlipidemia, admission blood pressure, admission glucose, admission platelet count, and international normalized ratio (INR), stroke etiology by Trial of ORG 10172 in Acute Stroke Treatment (TOAST)^11^ criteria, thrombolysis in cerebral infarction 2b/3 reperfusion status, NIHSS score at 24 hours and mRS score at 90 days by means of telephonic or personal interview done by mRS‐certified neurologists. Data were stored in local registries, extracted, anonymized, and pooled using a standardized form with pre‐specified parameters into a central database. Ethics committee (EK‐Nr. 1080/2018, Medical University Vienna) approved the study including the pooling of the anonymized data from the respective local registries. Additional local ethical approval for the extraction and transfer of anonymized data was not required.

### Control Group

The control group consisted of WUS/SUO patients not undergoing IVT extracted from The Acute Stroke Registry and Analysis of Lausanne database^12^ and from the Austrian Stroke Registry^13^, ^14^ using the same inclusion and exclusion criteria as mentioned above as a filter.

### Imaging

Admission CT scan readings were performed using the eASPECTS software (https://www.brainomix.com) at 3 of the 5 study sites and by dedicated neuroradiologists using the ASPECTS^10^ at the remaining 2 sites. For the control group, the ASPECTS were extracted from the respective registries. CT scans at 24 hours were analyzed by an independent masked radiologist at local sites and categorized into no hemorrhagic transformation, any intracranial hemorrhage, hemorrhagic transformation type 1 (HT1) and type 2 (HT2) and parenchymal hemorrhage type 1 (PH1) and type 2 (PH2) according to ECASS study.^15^

### Primary Outcome and Secondary Outcome Measures

The primary outcome was the occurrence of symptomatic intracranial hemorrhage (SICH) defined as any new intracranial hemorrhage causing clinical deterioration ≥4 NIHSS points according to ECASS3 criteria.^1^ For the control group, the SICH data were extracted from the respective registries. Secondary outcomes included NIHSS improvement ≥4 points in the first 24 hours,^16^ mRS score 0 or 1 at 90 days and mRS shift between pre‐stroke mRS and mRS at 90 days.

## Statistical Analysis

All statistics were performed using statistical software SPSS 24.0 (SPSS Inc., Chicago, USA). Distribution of the data was visualized using histograms and tested using the one‐sample Kolmogorov–Smirnov test. Results are presented as mean, range, and standard deviation (SD) for normally distributed variables and as median, range, and interquartile range (IQR) for non‐normally distributed factors. Data for categorical variables are summarized in absolute frequencies (n) and percentages. Two patient groups were created based on IVT administration. The IVT group consisted of patients extracted from the prospective multicenter registry. The control group consisted of patients not undergoing IVT extracted from Acute Stroke Registry and Analysis of Lausanne database and Austria Stroke Registry using identical inclusion and exclusion criteria as a filter. Propensity score was used to adjust for treatment effects of measured confounders. Propensity score matched analysis was performed using the SPSS build‐in algorithm. Pre‐defined confounders included age, admission NIHSS score, pre‐stroke mRS score, admission ASPECTS, admission blood pressure, admission glucose level, admission platelet count and INR, stroke etiology by TOAST criteria, and thrombectomy. The procedure first tries exact matches 1:1 followed by fuzzy matches if unsuccessful. Caliper was set to 0.1. Baseline variables were compared before and after matching to check for reduction of bias. Adding time metrics into the propensity‐matching procedure resulted in increased bias and was therefore omitted. Following the matching procedure, a multivariable logistic regression model was performed to adjust for relevant outcome confounders (age, premorbid mRS, admission NIHSS score, ASPECTS, stroke etiology by TOAST, thrombolysis in cerebral infarction 2b/3 reperfusion status, last seen well‐door times, and center). For the sensitivity analysis matched pairs were simply dropped if both (case and control) underwent thrombectomy. This approach was adopted because of the fact that the resulting sample size would be too small to fit a propensity score model with the given number of covariates. The threshold for statistical significance was set to *P*<0.05. The analysis protocol was finalized before the start of data analysis. The study has been registered at Clinicaltrials.gov (NCT03634748) after the start of the study.

## Results

**Figure 1:**
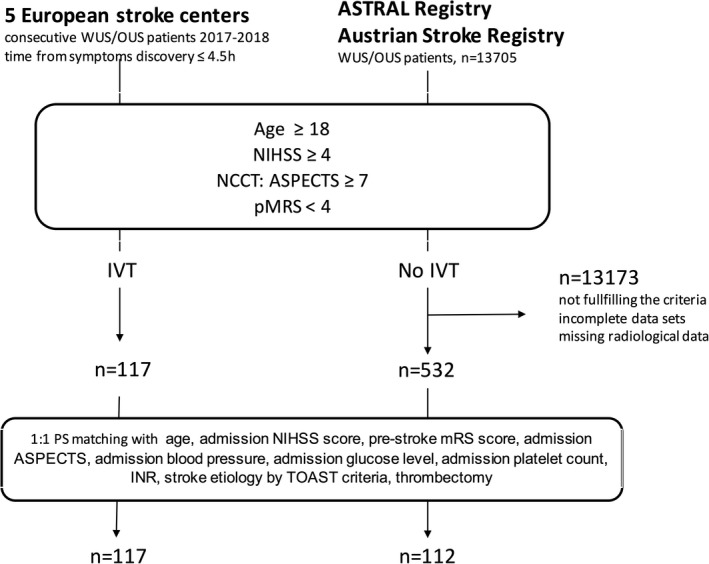
Flowchart with simplified inclusion criteria showing the study population. ASTRAL indicates The Acute Stroke Registry and Analysis of Lausanne database; ASPECTS, Alberta Stroke Program Early Computerized Tomography Score; INR, international normalized ratio; IVT, intravenous thrombolysis; mRS, modified Rankin Score; NCCT, non‐contrast computed tomography; NIHSS, National Institute of Health Stroke Scale; OUS, unknown onset stroke; pMRS, premorbid modified Rankin Score; PS, propensity score; TOAST, Trial of ORG 10172 in Acute Stroke Treatment; WUS, wake‐up stroke.

In total, 117 WUS/SUO patients undergoing NCCT‐based IVT fulfilled the inclusion criteria and for the pooled control group (WUS/SUO patients not undergoing IVT), we identified 532 patients with full respective data sets from the Acute Stroke Registry and Analysis of Lausanne database (n=1171 for WUS/SUO) and from the Austrian Stroke Registry (n=12 534 for WUS/SUO) filtered by the same inclusion and exclusion criteria. After propensity score matching, 117 IVT patients were compared with 112 propensity matched control patients, see Figure 1. The median NIHSS score was 10 and the median ASPECTS was 10 in both group, respectively. 29 (24.8%) IVT patients and 24 (21.4%) controls underwent additional endovascular thrombectomy. Age, sex, risk factors, stroke etiology, clinical and laboratory characteristics were well balanced between the groups, see Table 2. In the IVT group any new intracranial hemorrhage was present in 24 (20.5%), HT1 in 6 (5.1%), HT2 in 3 (2.6%), PH1 in 6 (5.1%) and PH2 in 1 (0.9%). In the control group any new intracranial hemorrhage was present in 15 (13.4%), HT 1 in 7 (6.3%), HT2 in 2 (1.8%), PH1 in 4 (3.6%) and PH2 in 2 (1.8%). The *P* values for the comparisons were *P*=0.16, *P*=0.78, *P*=1, *P*=0.75, and *P*=0.61, respectively.

**Table 2:** Comparison of Baseline Characteristics Between the IVT Patients and Control Group

Characteristics	IVT, n=117	Controls, n=112	*P* Value
Age, y, mean, (range, SD)	73.4 (38–95, 11.7)	69.9 (28–98, 14.9)	0.1
Sex, men, n (%)	65 (55.6)	60 (53.6)	0.8
LSW to door, min., median (range, IQR)	574 (300–1215, 281)	738 (354–1430, 564)	0.001
SDT to door, min., median (range, IQR)	85 (13–270, 62)	170 (17–1350, 323)	0.001
Wake‐up stroke, n (%)	104 (88.9)	96 (85.7)	0.5
Pre‐stroke mRS score, n (%)			
0	76 (65)	60 (53.6)	0.3
1	16 (13.7)	23 (20.5)	
2	10 (8.5)	14 (12.5)	
3	15 (12.8)	15 (13.4)	
Previous stroke/TIA, n (%)	25 (21.4)	33 (29.5)	0.2
Hypertension, n (%)	99 (84.6)	96 (85.7)	0.8
Diabetes mellitus, n (%)	30 (25.6)	38 (33.9)	0.2
Atrial fibrillation, n (%)	46 (39.3)	37 (33)	0.3
Coronary artery disease, n (%)	28 (23.9)	21 (18.8)	0.4
Hyperlipidemia, n (%)	75 (64.1)	80 (71.4)	0.3
Admission NIHSS score, median (range, IQR)	10 (4–32, 11)	10 (4–36, 12)	0.1
Admission glucose, median (range, IQR)	125 (75–430, 63)	150 (80–268, 68)	0.2
Admission BP systolic, median (range, IQR)	150 (105–250, 35)	153 (80–220, 44)	0.3
Admission BP diastolic, median (range, IQR)	80 (30–130, 18)	83 (40–130, 25)	0.2
Admission platelet count, median (range, IQR)	231 (111–511, 83)	230 (76–998, 116)	0.3
Admission INR, median (range, IQR)	1.0 (0.81–1.7, 0.1)	1.0 (0.8–1.7, 0.1)	0.6
ASPECTS, n (%)			
10	68 (58.1)	69 (63.3)	0.1
9	24 (20.5)	12 (11)	
8	19 (16.2)	15 (13.8)	
7	6 (5.1)	13 (11.9)	
Additional thrombectomy, n (%)	29 (24.8)	24 (21.4)	0.6
TICI 2b/3, n (%)	27 (93.1)	22 (91.7)	1.0
Stroke etiology, n (%)			
Cardioembolic	53 (47.3)	60 (52.6)	0.8
Small vessel disease	14 (12.5)	16 (14)	
Large vessel disease	14 (12.3)	14 (12.5)	
Other	6 (5.3)	9 (8)	
Cryptogenic	18 (15.8)	22 (19.6)	

ASPECTS indicates Alberta Stroke Program Early Computerized Tomography Score; BP, blood pressure; INR, international normalized ratio; IQR, interquartile range; LSW, last seen well time; mRS, modified Rankin Scale; NIHSS, National Institute of Health Stroke Scale; SDT, symptom discovery time; TIA, transient ischemic attack; TICI, thrombolysis in cerebral infarction scale grade.

### Primary End Points

SICH according to ECASS3 criteria occurred in 4 (3.4%; 95% CI 0.9%–8.5%) patients undergoing IVT as compared with 1 (0.9%, CI 0%–4.9%) patients in the control group (odds ratio (OR) 3.8, 95% CI 0.4–34.7, *P*=0.2). After adjustment (age, premorbid mRS, admission NIHSS score, ASPECTS, stroke etiology by TOAST, thrombolysis in cerebral infarction 2b/3 reperfusion status, last seen well‐door times, and center) the adjusted OR (aOR) for SICH was 7.9, CI 0.65–96, *P*=0.1, see Table 3.

**Table 3:** Odds Ratios for Associations Between IVT and Outcome Measures

Outcome	Crude OR	95% CI	*P* Value	Adjusted OR^a^	95% CI	*P* Value
SICH ECASS3	3.8	0.4 to 34.7	0.2	7.9	0.65 to 96	0.1
NIHSS ≥4 change	4.7	2.6 to 8.3	<0.001	5.8	3.0 to 11.2	<0.001
mRS 0 to 1	1.9	1.2 to 3.5	0.03	1.94	1.001 to 3.76	0.05
mRS shift	0.48	0.25 to 0.9	0.02	0.47	0.22 to 0.99	0.05

mRS, modified Rankin Scale; NIHSS, National Institute of Health Stroke Scale; OR, odds ratio; SICH, symptomatic intracranial hemorrhage according to ECASS3 study definition.

a Adjusted for age, premorbid mRS, admission NIHSS score, Alberta Stroke Program Early Computerized Tomography score, stroke etiology by Trial of ORG 10172 in Acute Stroke Treatment, thrombolysis in cerebral infarction 2b/3 reperfusion, last seen well‐door times, and center.

### Secondary End Points

**Figure 2:**
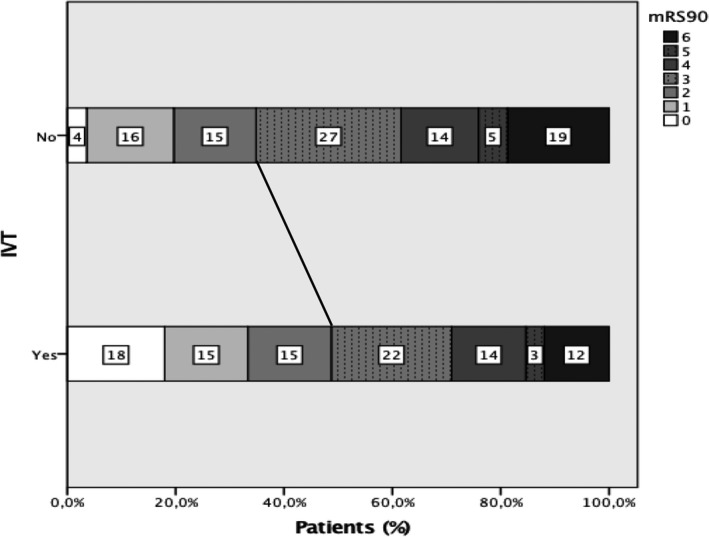
Modified Rankin Scale at 90 days in IVT patients and controls. IVT indicates intravenous thrombolysis, mRS, modified Rankin Score.

Improvement in NIHSS by ≥4 points between admission and 24 hours thereafter occurred in 67 (57.3%) of IVT patients as compared with 25 (22.3%) in controls (OR 4.7, CI 2.6–8.3, <0.001). After adjustment, the aOR was 5.8, CI 3.0 to 11.2, *P*<0.001. At 3 months, a modified Rankin Scale score of 0 or 1 was present in 39 (33.3%) IVT patients versus 23 (20.5%) controls (OR 1.9, CI 1.2–3.5, *P*=0.03), see Figure 2. After adjustment, the aOR was 1.94, CI 1.001 to 3.76, *P*=0.05. A shift in the modified Rankin Scale between admission and 3 months was present in 82 (70.1%) IVT patients versus 93 (83%) controls (OR 0.48, CI 0.25–0.9, *P*=0.02). After adjustment, the aOR was 0.47, CI 0.22 to 0.99, *P*=0.05, see Table 3.

### Sensitivity Analysis After Excluding Patients Who Underwent Thrombectomy

Eighty‐eight patients underwent IVT only and 88 controls had no recanalization therapy at all.

The median admission NIHSS score was 8 (range 4–28) in the IVT group and 10 (range 4–36) in the control group (*P*=0.03). The median ASPECTS was 10 and 10, respectively. Age, premorbid mRS and etiology by TOAST showed non‐significant differences between the groups (*P*=1, *P*=0.2 and *P*=0.2), see Table 4. SICH occurred in 2 of 88 IVT patients (2.3%; 95% CI 0.3%–8.1%) as compared with 0 of 88 controls (0%, CI 0%–4.2%). Improvement in NIHSS by ≥4 points between admission and 24 hours thereafter occurred in 42 (48.8%) of IVT patients as compared with 20 (23.2%) in controls (OR 3.1, CI 1.6–5.9, *P*=0.001). After adjustment (age, premorbid mRS, admission NIHSS, ASPECTS, stroke etiology by TOAST, last seen well times, and center), the aOR was 4.2, CI 1.9 to 9.0, *P*<0.001. At 3 months, a modified Rankin Scale score of 0 or 1 was present in 32 (37.2%) IVT patients versus 21 (24.4%) controls (OR 1.8, CI 0.95–3.51, *P*=0.07). After adjustment, the aOR was 1.3, CI 0.6 to 2.8, *P*=0.4. Shift in the modified Rankin Scale between admission and 3 months was present in 56 (65.1%) IVT patients versus 93 (83.7%) controls (OR 0.39, CI 0.19–0.78, *P*=0.008). After adjustment, the aOR was 0.46, CI 0.2 to 1.05, *P*=0.06.

**Table 4:** Comparison of Baseline Characteristics Between the IVT Patients and Control Group After Excluding Patients Undergoing Thrombectomy

Characteristics	IVT, n=88	Controls, n=88	*P* Value
Age, y, mean, (range, SD)	73.4 (38–95, 12)	73.2 (38–98, 12)	1
Sex, men, n (%)	49 (55.7)	50 (56.8)	1
LSW to door, min., median (range, IQR)	620 (300–1215 272)	772 (354–1430, 496)	0.001
SDT to door, min., median (range, IQR)	85 (18–270, 59)	212 (17–1350, 387)	<0.001
Wake‐up stroke, n (%)	80 (90.9)	79 (89.8)	1
Pre‐stroke mRS score, n (%)			
0	57 (64.8)	50 (56.8)	0.5
1	11 (12.5)	18 (20.5)	
2	9 (10.2)	11 (12.5)	
3	11 (12.5)	9 (10.2)	
Previous stroke/TIA, n (%)	21 (23.9)	26 (29.5)	0.5
Hypertension, n (%)	73 (83)	73 (83)	1
Diabetes mellitus, n (%)	25 (28.4)	34 (38.6)	0.2
Atrial fibrillation, n (%)	33 (37.5)	31 (35.2)	0.9
Coronary artery disease, n (%)			
Hyperlipidemia, n (%)			
Admission NIHSS score, median (range, IQR)	8 (range 4–28, 9)	10 (4–36, 14)	0.03
Admission glucose, median (range, IQR)	122 (75–430, 75)	131 (80–198, 25)	0.4
Admission BP systolic, median (range, IQR)	150 (105–205, 33)	153 (107–213, 51)	0.6
Admission BP diastolic, median (range, IQR)	80 (30–130, 20)	85 (55–116, 28)	0.2
Admission platelet count, median (range, IQR)	242 (111–511, 86)	234 (130–999, 119)	0.8
Admission INR, median (range, IQR)	1 (0.8–1.4, 0.1)	1 (0.8–1.3, 0)	0.3
ASPECTS, n (%)			
10	57 (64.8)	59 (67)	0.2
9	17 (19.3)	9 (10.2)	
8	10 (11.4)	10 (11.4)	
7	4 (4.5)	10 (11.4)	
Stroke etiology, n (%)			
Cardioembolic	44 (55)	36 (45)	0.2
Small vessel disease	16 (18.6)	14 (15.9)	
Large vessel disease	11 (12.8)	9 (10.2)	
Other	4 (4.7)	8 (9.4)	
Cryptogenic	11 (12.8)	21 (23.9)	

ASPECTS indicates Alberta Stroke Program Early Computerized Tomography Score; BP, blood pressure; INR, international normalized ratio; IQR, interquartile range; LSW, last seen well time; mRS, modified Rankin Scale; NIHSS, National Institute of Health Stroke Scale; SDT, symptom discovery time; TIA, transient ischemic attack.

## Discussion

The primary aim of this study was to examine safety of IVT in WUS/SUO selected using NCCT and ASPECTS. As expected, we found slightly more SICH in the IVT group as compared with controls. The observed rate of 3.4% is comparable with rates in WUS studies using advanced MR or CT‐perfusion imaging (WAKE‐UP, EXTEND) and in a real‐world experience with IVT in WUS.^4^, ^5^, ^17^ Thus, our observation suggests acceptable safety of IVT in WUS/SUO with normal or near‐normal NCCT appearance. This is also in line with previous reports on the use of NCCT in the WUS selection for IVT. Barreto et al reported none and Anaissie et al reported only 1 SICH in 40 and 46 patients undergoing NCCT‐based thrombolysis for WUS, respectively.^7^, ^8^

Secondary aims included established measures of IVT efficacy. As compared with matched controls we observed more IVT patients improving at 24 hours, reaching more often favorable functional outcome at 3 months and experiencing less shift in the mRS score indicating possible clinical efficacy. Importantly, the sensitivity analysis after excluding patients undergoing thrombectomy seem to indicate that the positive efficacy signals may not be driven by effects of thrombectomy alone. After adjustment some end points missed in the later analysis the statistical threshold for significance; this seems, however, to be because of the reduced sample size and consequent lack of statistical power.

Previous studies describing radiological and clinical features of WUS suggested that the putative symptom onset is most likely during the morning hours and just before awakening, indicating that WUS patients may have salvageable tissue at hospital arrival.^18^, ^19^, ^20^, ^21^, ^22^ The high percentage of WUS (89%) in our study seems to underline this hypothesis. In analogy to the time‐based concept of IVT in known onset stroke without penumbra imaging, we hypothesize that WUS patients reaching hospital within 4 to 4.5 hours of awakening may benefit from IVT using the same imaging paradigm, ie, NCCT.

Time delays from last seen well and symptom discovery in the IVT group and controls in our study were markedly different. This may be because of the fact that the main reason for excluding WUS patients from IVT in the control group was the time from symptom discovery >4.5 hours. However, we suggest that this difference should not bias the safety nor the efficacy results as the outcome measures in the control group (thus not receiving IVT) are not time dependent. Importantly, after adjustment for time metrics in the multivariate regression models, the results remained grossly unchanged.

Limitations of our study have to be mentioned. Despite being based on a rigorously managed prospective registry, the design of the study has to be acknowledged as retrospective, including all the associated limitations. CT readings were not centralized as well as there was not a uniform use of eASPECTS software across the centers. The assessment of NIHSS and mRS scores at 3 months was performed at each center separately. This may have introduced bias into the efficacy analysis albeit assessed by NIHSS‐ and mRS‐certified neurologist only. Finally, and most importantly, possible bias by indication and bias by low sample size has to be considered. Data on WUS patients excluded from IVT were not collected precluding assessment of the bias by indication. Low sample size precludes definitive statements on safety and efficacy as with this number of observations the study remains clearly underpowered. Reported *P* values are not adjusted for multiple testing. Given the sample size, we considered the analysis to be exploratory, thus without adjusting *P* values. Thus, the interpretation of our results has to be made with caution, considering the above‐mentioned limitations and should be used for hypothesis generating only. On the other hand, our observation seems to be to date the largest experience on NCCT‐based IVT in WUS/SUO and provides a propensity score–matched control group mimicking randomization. It suggests no excess in symptomatic hemorrhage and sets signals of efficacy.

## Conclusions

To date, WUS/SUO represent an undertreated subgroup of acute stroke patients. Despite the fact that advanced neuroimaging has been recently proven to be safe and effective for IVT selection in this population, it is by far not widely available. Magnetic resonance imaging or CT‐perfusion imaging might perform superiorly in selecting WUS/SUO patients for IVT in means of safety and efficacy. However, a simple, widely available, and pragmatic NCCT‐based selection approach may eventually offer IVT indication to those who would otherwise be excluded from this treatment because of missing advanced imaging facilities. Our study suggests that an acceptable safety and eventually efficacy can be achieved by using solely NCCT for selecting IVT candidates in the WUS/SUO population. Randomized proof of this concept or a head‐to‐head comparison with advanced neuroimaging is highly warranted.

## Disclosures

None.
